# Thirty-Third Annual Meeting of the Ohio State Dental Society, Held at Columbus, Ohio, Dec. 5, 6, 7, 1899

**Published:** 1900-01-15

**Authors:** J. R. Owens, Everett M. Cook, Otto Arnold


### THIRTY-THIRD ANNUAL MEETING OF THE OHIO STATE DENTAL SOCIETY, HELD AT COLUMBUS, OHIO, DEC. 5, 6, 7, 1899.

### PRESIDENT'S ADDRESS, BY L. P. BETHEL, D.D.S.

To-day we meet again in annual session with every indication of another profitable meeting.

During the past few years the Ohio State Dental Society has received a great impetus through the faithful work of its members, and there is no reason why this society should not continue one of the best in America. We have among its members the requisite talent, energy and disposition. Harmony, willingness on the part of every member to do his duty, and faithful work, are the essentials for its maintenance.

#### THE YOUNG MEN.

We have been impressed with the numbers of young men coming into our society. Welcome them; give them encouragement; set them to work, for they are the ones upon whom the perpetuation of the society depends.

LOCAL DENTAL SOCIETIES.

There are a number of very active local dental societies throughout the State, and they no doubt have an influence on the State society. They not only benefit every member personally, but they unite men professionally and bring about a union that is most desirable. Let us have more of these organizations.

DENTAL LEGISLATION.

There is great need of better dental legislation in Ohio. The present law is but a poor excuse, wholly inadequate for the demands, and should be remedied. Perhaps no one realizes this fact more fully than the members of the Board of Dental Examiners.

There seems to be nothing in the way of having a good law passed, for there is harmony in the profession and surely every right-minded dentist should be willing to aid its passage.

Other States are ahead of Ohio in this respect and it is hoped that our legislation committee can succeed, this session of the Legislature, in securing a just law that will protect both the public and the profession from impostors and detestable quacks.

EXEMPTION FROM JURY DUTY.

While on the subject of legislation, we might mention that another desirable law for dentists would be that of exemption from jury duty. It is always a great sacrifice and loss to the dentist whenever compelled to serve in this capacity, and, like the physician, he should be exempt. His reasons are of the best; they are tangible and should receive recognition by our legislators.

APPOINTMENT OF DENTISTS TO STATE INSTITUTIONS.

The teeth play so important a part in the animal economy their salvation is a matter of great importance for the welfare and health of every individual, and attention should be given the teeth, especially those diseased, as well as to other physical infirmities.

Physicians are appointed by the State for State institutions. Why should not dentists also be appointed to them ?

I understand that steps in this direction have already been taken in Georgia, and that a dentist has been appointed to the Georgia Insane Asylum at Macon.

HYGIENE IN THE SCHOOLS.

Dental and oral hygiene should be taught in our public schools, especially in the primary grades.

A year ago the Superintendent of the Cleveland city schools became interested in this matter, and a course of instruction in oral hygiene was instituted. From reports it seems to have been a successful effort. The schools of Toledo are about to adopt something of this sort, and smaller places are becoming interested.

It might be well for this society to appoint a standing committee on hygiene, and let them prepare a scheme for instruction and recommend its adoption by teachers in the public schools of the State.

DENTISTS IN THE ARMY AND NAVY.

Another effort will be made to secure appointments of dentists in the army and navy of the United States. Aside from the committee of legislation, the National Dental Association has, by vote, appointed a committee at large consisting of one member of the National Association from each State, to act in conjunction with any State or local committees in furthering this project. As this society has already a committee favoring Corps of Dental Surgeons for the army and navy, I would suggest that this committee be continued and increased if deemed advisable by this body.

SCIENTIFIC WORK—COMMITTEE ON INVESTIGATION.

The importance of proof, through investigation, is apparent to every one, and it might stimulate research work if the society had a committee on investigation and research. If several members could conduct a series of experiments along the same lines, and at the following meeting give the comparative results of this search work, it seems to me that great good would come from it. We have many members capable of carrying on such investigations, and the results would be beneficial to investigators as well as to the members of the society in general.

### COMMITTEE ON RECORDS.

As it is of great importance that all records of this society be complete and that they be properly inscribed, I would suggest a standing committee whose duty it shall be to examine the records each year and see that they are complete.

### DENTAL CONGRESS IN 1902.

Some months ago the Toledo Dental Society passed the following resolutions:

WHEREAS, The Ohio Centennial is to be held at Toledo, Ohio, in 1902, and naturally many of our profession will be in attendance; and

WHEREAS, The Toledo Dental Society being at the home of this great enterprise, we should agitate the holding of a Dental Congress some time during the centennial session; therefore, be it

*Resolved,* That our President appoint a committee of three to bring this matter before the next meeting of the Ohio State Dental Society.

I merely call your attention, at the present time, to this matter, hoping that if it be formally presented to this body, you will give it due consideration and take such action as the members deem appropriate.

In conclusion, I will state that I have purposely made this address a short one, desiring mainly to submit these few suggestions to you. The Executive, Clinic and Arrangement committees have worked assiduously and have prepared an excellent program for our meeting. I

trust that you will all take active part that the best good may be gotten from the subjects treated.

REPORT OF COMMITTEE ON PRESIDENT'S ADDRESS.

Your committee, appointed to report on the suggestions contained in the president's address, beg leave to submit the following:

We most heartily concur in the sentiments of the address as a whole, but would emphasize the importance of certain suggestions therein specified and recommend for immediate action by this body:

First. The appointment by the president of a committee of nine (9) to formulate plans for a Dental Congress to be held in Toledo in 1902, and report at the next annual meeting.

Second. The appointment of a committee of five (5) by the president, of which Dr. W. A. Price shall be chairman, to outline or formulate a system for a course of study in Dental Hygiene, and a method by which it may be introduced into the public schools of the State.

We furthermore recommend that the appointment of dentists in State institutions and the exemption of dentists from jury duty be referred to the legislative committee; also the continuation of standing committee on matters pertaining to employment of dentists in the army and navy.

J. R. Owens,
Everett M. Cook,
Otto Arnold.

